# Positive mental health in outpatients: comparison within diagnostic groups

**DOI:** 10.1186/s12888-016-1113-1

**Published:** 2016-11-18

**Authors:** Rajeswari Sambasivam, Janhavi Ajit Vaingankar, Siow Ann Chong, Edimansyah Abdin, Anitha Jeyagurunathan, Lee Seng Esmond Seow, Shirlene Pang, Mythily Subramaniam

**Affiliations:** Research Division, Institute of Mental Health, Singapore, Singapore

**Keywords:** Positive mental health, Well-being, PMH, Multi-ethnic, Outpatients

## Abstract

**Background:**

Positive mental health (PMH) supplements the definition of mental health which is not just the mere absence of mental illness. It encompasses an individual’s social, emotional and psychological well-being. This cross-sectional study examines the PMH levels in a multi-ethnic outpatient population and the socio-demographic correlates of PMH across the various diagnostic groups. In addition comparisons with the general population were conducted.

**Methods:**

Outpatients with schizophrenia spectrum, depressive or anxiety disorders seeking treatment at a tertiary psychiatric care hospital were included in the study sample. All respondents completed the PMH instrument. Independent t-tests and ANOVA with Bonferroni post-hoc tests were used to establish differences between the PMH levels and domains.

**Results:**

Three hundred and sixty outpatients with a mean age of 39.2 years were included in the study. 52.5% were younger adults (21–39 years). There were slightly more males (50.8%) and 56.1% of the sample was unemployed. PMH scores differed between the patient and general populations. There were significant associations of the PMH domains with socio-demographic variables such as age, ethnicity, gender and education status in the patient population.

**Conclusions:**

PMH can be viewed as a protective factor of mental illnesses. As such it is critical that mental health professionals examine the domains of PMH in individuals with mental illnesses. This will in turn allow them to develop coping strategies that can look into focusing on emotional, psychological and social well-being appropriately to allow these individuals to thrive.

## Background

Mental health, according to the World Health Organisation [1], is a state of integral physical, mental and social well-being, whereby the presence of disease is overcome, while on the other hand, it also includes the capability of leading or living an economically and socially productive life. Just as positive affect is not the opposite of negative affect, well-being is not just the absence of mental illness [2]. Keyes [3] proposed a two continua model which holds that mental illness and mental health are related but distinct dimensions: one continuum indicates the presence or absence of mental health and the other indicates the presence or absence of mental illnesses. The model gives rise to four possible states that an individual might experience- thriving/flourishing (with mental illness), thriving/flourishing (without mental illness), surviving/languishing (without mental illness), surviving/languishing (with mental illness) [4]. Extending on this model Ryan & Deci [5] discuss that most researchers had structured well-being into two broad domains: one describing happiness (hedonic well-being), and the other human potential (eudaimonic well-being) [4, 6]. The concept of subjective well-being is primarily rooted in the hedonistic concept of well-being, “by which well-being is operationally defined by a high level of positive effects, a low level of negative effects and high degree of life satisfaction” [6]. From the eudaimonic perspective, subjective reports of people on the sum of current feelings at a specific time do not indicate that they are psychologically or socially well, hence implying subjective happiness cannot be equated with well-being [5]. Psychological well-being, which lies within the eudaimonic concept, comprises six basic dimensions [7]: self-acceptance, personal growth, autonomy, relationships with others, environmental mastery and purpose in life.

Positive mental health (PMH) incorporates a combination of these aspects and it serves as a strong protective factor against mental illness. Mental disorders and PMH are two overlapping and interrelated components of mental health [8]. Factors such as self-esteem, emotional resilience, positive thinking, problem-solving skills, social skills, stress management skills and feelings of mastery are aligned with aspects of PMH. These are also recognized as individual protective factors in mental disorder prevention. For this reason, preventive interventions which aim to strengthen protective factors overlap largely with PMH. Both risk and protective factors can be individual, family-related, socio-economic and environmental in nature. Risk factors are related to an increased likelihood of onset, greater severity and prolongation of major health problems. Conditions that improve people’s resistance to risk factors and disorders are referred to as protective factors; which have been defined as factors that “modify, ameliorate or alter a person’s response to some environmental hazard that predisposes to a maladaptive outcome” [9]. Multiple risk factors and the lack of protective factors predispose individuals to increased vulnerability that can potentially lead to a mental health problem. Mental health preventive interventions aim to counteract risk factors and reinforce protective factors in order to prevent human mental dysfunction. Thus when the individual protective factors (individual domains of PMH) that have more influence on the development of mental disorders and mental health are duly managed, PMH will impose a greater preventive effect [8].

Many studies have indicated that emotional, psychological and social well-being together form an individual’s PMH [3], taking the hedonic and eudaimonic perspectives of well-being into consideration. Thus, to completely comprehend an individual’s PMH, their emotional, psychological, as well as social well-being should be measured. It is therefore important to assess the various domains of PMH in individuals with mental illnesses, as it will aid mental health professionals to better understand the individuals’ needs and assist in reducing symptoms by improving their mental health.

PMH has been assessed in a multi-ethnic adult community based in Singapore in a different study previously [10]. This current study aimed 1) to examine the scores of PMH and its domains among people with mental illness (namely three diagnostic groups: schizophrenia spectrum disorders, depressive disorders and anxiety disorders), 2) to explore socio-demographic and clinical correlates of PMH and 3) to further observe the relation of the mean scores to that of the general population by using one-sample t-tests.

## Methods

### Sample population

The detailed survey methodology is described elsewhere [11]. The PMH survey was a cross sectional study conducted on a convenience sample of adult outpatients with mental illnesses seeking treatment at the Institute of Mental Health in Singapore. Patients who were Singapore citizens and Permanent Residents of Chinese, Malay and Indian ethnicities and aged between 21 and 65 years were considered eligible for the study. In addition, they had to have a clinical diagnosis of schizophrenia spectrum disorders, depressive or anxiety disorders and this was confirmed with their attending physicians. The physicians at the Institute of Mental Health were employing the ICD-9 during the duration of this study recruitment which was between January 2014 and May 2015. All respondents provided informed consent and were English literate. Of the 360 subjects, 207 were referred by doctors, 84 were self-referred and 69 were referred by other health professionals. Posters informing patients of the ongoing study were placed in the clinics along with contact information of the study team members and this aided in the self-referrals by the patients. Psychiatrists and other healthcare professionals (nurses, psychologists, medical social workers and case managers) were requested to refer their patients for the study. A quota sampling plan was put in place to ensure adequate representation by diagnosis, age, gender and ethnic groups.

Ethics approval was obtained from the Domain Specific Review Board of the National Healthcare Group, Singapore.

### General population sample

The PMH instrument was previously utilized to measure the level of PMH in a multi-ethnic community-based population as part of the Singapore Mental Health Study (SMHS). The survey methodology has been described in detail in another paper [10]. The study was conducted between April 2010 and February 2011. Respondents (*n* = 404) were recruited randomly through household level purposive sampling. Singapore citizens and Permanent Residents belonging to Chinese, Malay or Indian ethnicity, aged 21 to 65 years and literate in English were included in the study. Participants were requested to complete this questionnaire within 3 days at their own convenience and to place them in a sealed envelope to ensure confidentiality and reduce social desirability bias. The mean scores reported from this study have been utilized for comparison purposed in this current study.

### Data collection & measures

Data was collected on two separate data collection forms (Questionnaire I and Questionnaire II). Questionnaire I was used to obtain socio-demographic information of the participants such as age, gender, ethnicity, educational level, marital and employment status. It was administered by an interviewer and was completed upon enrolment.

Questionnaire II consisted of the PMH instrument which is a validated self-report measure that has previously been used to establish PMH levels in a large multi-ethnic population sample in Singapore [10]. This is a 47-item instrument that encompasses six subscales: general coping (GC) (nine items), emotional support (ES) (seven items), spirituality (seven items), interpersonal skills (IS) (nine items), personal growth and autonomy (PGA) (ten items), and global affect (GA) (five items). For the first five subscales, participants were requested to indicate how much each item describes them on a scale from 1 to 6 (1- ‘Not at all like me’ to 6- ‘Exactly like me’). For the ‘global affect’ subscale, participants were requested to indicate ‘how often over the past four weeks they felt – calm, peaceful, relaxed and enthusiastic’ using a 5-point response scale (1- ‘Never or very rarely’ to 5- ‘Very often or always’). Domain-specific scores were calculated by summing the scores of the respective items and dividing by the number of items in each domain, likewise, for the total PMH score.

Questionnaire II was self-administered and participants had to complete the PMH instrument. Participants were requested to complete this questionnaire within 3 days by themselves at their own convenience and instructed to place them in a sealed envelope to ensure confidentiality and reduce social desirability bias. The questionnaires were then collected by interviewers.

### Statistical analyses

Statistical analyses were performed using IBM SPSS, version 23. Independent t-tests and ANOVA with Bonferroni post-hoc tests were used to establish differences in mean scores for total PMH and its specific domains by socio-demographic and patient subgroups. To adjust for confounding effects, multiple linear regression analysis was used to determine the associations between each PMH domains and socio-demographic variables. All statistically significant differences were evaluated at *p* value < 0.05 using 2-sided tests.

## Results

The study population comprised 360 outpatients with a mean age of 39.2 (SD = 11.1) years. Socio-demographic and clinical characteristics of the respondents are shown in Table 1. The sample had a slightly higher proportion of those aged 21–39 years (52.5%), males (50.8%) who were unemployed (56.1%). Majority were Chinese, single and had college or pre-university education.

**Table 1:** Socio-demographic & clinical characteristics of the sample (*n* = 360)

		Number	Percent
Age group	21–39 years	189	52.5
	40–65 years	171	47.5
Gender	Males	183	50.8
	Females	177	49.2
Ethnicity	Chinese	145	40.3
	Malay	106	29.4
	Indian	109	30.3
Marital status	Single	200	55.6
	Married	108	30.0
	Separated/Divorced/Widowed	52	14.4
Education	Some formal/primary	39	10.8
	Secondary/Junior College/Pre University	235	65.3
	Vocational	41	11.4
	Tertiary	37	10.3
	Post grad	8	2.2
Employment status	Unemployed	202	56.1
	Employed	157	43.6
Diagnostic Group	Schizophrenia spectrum disorders	142	39.4
	Depressive disorders	139	38.6
	Anxiety disorders	79	21.9

### Socio-demographic & clinical differences in PMH total and domain-specific scores

The mean (SD) of the total PMH and domain-specific scores for the overall sample and by socio-demographic and diagnostic groups are tabulated in Table 2. Significant differences were observed in total PMH and domain scores across the socio-demographic variables. The total PMH score (*P =* 0.002) and domains, general coping (*P =* 0.001), spirituality (*P =* 0.001) and personal growth and autonomy (*P <* 0.001) scores were found to be significantly higher among those aged between 40 and 65 years. There were gender differences for emotional support whereby females (*P =* 0.010) had a significantly higher score as compared to men. The spirituality domain score was significantly higher among respondents belonging to the Malay or Indian ethnicity (*P <* 0.001). Those who were married reported higher scores in total PMH (*P =* 0.043) and domains, emotional support (*P =* 0.005), interpersonal skills (*P =* 0.013) and personal growth and autonomy (*P =* 0.026). The interpersonal skills (*P =* 0.030) domain score was significantly higher among the post-graduates in the education group. Differences in employment status existed for the total PMH score (*P =* 0.048), general coping (*P =* 0.005) and interpersonal skills (*P <* 0.001) domains, with those employed having significantly higher scores as compared to those unemployed. Significant differences were noted in total PMH and domain scores among the diagnostic groups as well. The total PMH score (*P* < 0.001) and domains, general coping (*P =* 0.001), emotional support (*P =* 0.001), personal growth and autonomy (*P =* 0.007) and global affect (*P <* 0.001) were significantly higher among those diagnosed with schizophrenia spectrum disorders as compared to those with depressive disorders. Similarly, significant higher total PMH (*P* < 0.001), general coping (*P =* 0.001) and global affect (*P* < 0.001) scores were observed in those with schizophrenia spectrum disorders as compared to those with anxiety disorders. Those diagnosed with anxiety disorders reported higher emotional support domain score (*P =* 0.001) as compared to those with depressive disorders. After adjusting with Bonferroni’s post hoc tests, the interpersonal skills domain score was significantly higher among respondents with Secondary/Junior College/Pre University education as compared to those with Some formal/Primary education (*P =* 0.030).

**Table 2:** Total PMH and domain scores across socio-demographic and clinical characteristics

	Total PMH Score		General coping		Emotional support		Spirituality		Interpersonal skills		Personal growth and autonomy		Global affect	
	Mean ± SD	*P*	Mean ± SD	*P*	Mean ± SD	*P*	Mean ± SD	*P*	Mean ± SD	*P*	Mean ± SD	*P*	Mean ± SD	*P*
Overall Sample	3.93 ± 0.95		3.84 ± 1.14		3.95 ± 1.33		3.98 ± 1.47		4.25 ± 1.04		3.94 ± 1.15		3.67 ± 1.17	
Age group														
21–39 years	3.79 ± 0.95	0.002	3.65 ± 1.19	0.001	3.92 ± 1.31	0.646	3.74 ± 1.54	0.001	4.16 ± 1.02	0.102	3.70 ± 1.19	<0.001	3.57 ± 1.18	0.075
40–65 years	4.10 ± 0.91		4.04 ± 1.04		3.99 ± 1.35		4.23 ± 1.35		4.34 ± 1.06		4.21 ± 1.03		3.79 ± 1.15	
Gender														
Male	3.88 ± 0.94	0.213	3.86 ± 1.13	0.670	3.78 ± 1.35	0.010	3.89 ± 1.49	0.257	4.21 ± 1.06	0.510	3.91 ± 1.14	0.613	3.61 ± 1.16	0.304
Female	4.00 ± 0.95		3.81 ± 1.13		4.13 ± 1.29		4.07 ± 1.45		4.29 ± 1.02		3.97 ± 1.15		3.73 ± 1.17	
Ethnicity														
Chinese	3.83 ± 0.92	0.089	3.70 ± 1.11	0.170	4.09 ± 1.30	0.056	3.54 ± 1.54	<0.001^a,b^	4.24 ± 0.97	0.674	3.79 ± 1.16	0.130	3.64 ± 1.12	0.868
Malay	4.10 ± 0.93		3.96 ± 1.19		4.02 ± 1.32		4.50 ± 1.23		4.32 ± 1.03		4.05 ± 1.12		3.72 ± 1.12	
Indian	3.92 ± 0.98		3.89 ± 1.10		3.70 ± 1.37		4.04 ± 1.42		4.20 ± 1.15		4.02 ± 1.14		3.67 ± 1.27	
Marital status														
Single	3.84 ± 0.98	0.043^c^	3.75 ± 1.19	0.289	3.86 ± 1.33	0.005^c,d^	3.86 ± 1.50	0.236	4.12 ± 1.05	0.013c	3.80 ± 1.19	0.026^c^	3.67 ± 1.17	0.498
Married	4.12 ± 0.88		3.95 ± 1.07		4.28 ± 1.22		4.12 ± 1.41		4.48 ± 0.95		4.15 ± 1.09		3.75 ± 1.16	
Separated/Divorced/Widowed	3.91 ± 0.89		3.92 ± 1.05		3.62 ± 1.42		4.11 ± 1.46		4.27 ± 1.11		4.03 ± 1.02		3.52 ± 1.18	
Highest education level														
Some formal/Primary education	3.71 ± 1.09	0.232	3.55 ± 1.13	0.114	3.67 ± 1.42	0.330	1.42 ± 0.23	0.344	3.80 ± 1.29	0.030^e^	3.80 ± 1.28	0.340	3.66 ± 1.23	0.472
Secondary/Junior College/Pre U	3.97 ± 0.94		3.90 ± 1.15		3.93 ± 1.32		4.08 ± 1.50		4.30 ± 1.02		3.98 ± 1.13		3.65 ± 1.14	
Vocational	4.00 ± 0.90		3.86 ± 1.12		4.22 ± 1.22		3.84 ± 1.46		4.22 ± 0.93		3.94 ± 1.13		3.93 ± 1.16	
Tertiary	3.78 ± 0.81		3.56 ± 0.97		4.03 ± 1.28		3.61 ± 1.42		4.30 ± 0.90		3.71 ± 1.13		3.48 ± 1.15	
Post Graduate	4.39 ± 1.15		4.38 ± 1.37		4.41 ± 1.87		4.18 ± 1.24		4.85 ± 0.95		4.54 ± 1.01		3.97 ± 1.78	
Current employment status														
Unemployed	3.85 ± 0.10	0.048	3.70 ± 1.17	0.009	3.87 ± 1.33	0.171	4.01 ± 1.45	0.621	4.08 ± 1.05	<0.001	3.85 ± 1.17	0.076	3.61 ± 1.17	0.201
Employed	4.05 ± 0.87		4.02 ± 1.06		4.06 ± 1.32		3.93 ± 1.50		4.46 ± 0.10		4.06 ± 1.10		3.76 ± 1.13	
Diagnostic group														
Schizophrenia spectrum disorders	4.24 ± 0.89	<0.001^f,g^	4.11 ± 1.08	0.001^f,g^	4.20 ± 1.24	0.001^f,h^	4.44 ± 1.23	<0.001	4.31 ± 1.06	0.498	4.16 ± 1.09	0.007^f^	4.23 ± 1.02	<0.001^f,g^
Depressive disorders	3.70 ± 0.92		3.66 ± 1.10		3.62 ± 1.36		3.78 ± 1.52		4.17 ± 1.07		3.75 ± 1.13		3.25 ± 1.07	
Anxiety disorders	3.80 ± 0.95		3.64 ± 1.21		4.11 ± 1.31		3.49 ± 1.57		4.29 ± 0.97		3.87 ± 1.21		3.41 ± 1.19	

Significant difference was set at *P* < 0.05 derived from independent *t*-test and one way ANOVA test

Bonferroni post hoc test:

^a^Significant difference between Malay vs Chinese

^b^Significant difference between Indian vs Chinese

^c^Significant difference between Married vs Single

^d^Significant difference between Married vs Separated/Divorced/Widowed

^e^Significant difference between Some formal/Primary education vs Secondary/Junior College/Pre U

^f^Significant difference between Depressive disorders vs Schizophrenia spectrum disorders

^g^Significant difference between Anxiety disorders vs Schizophrenia spectrum disorders

^h^Significant difference between Depressive disorders vs Anxiety disorders

### Correlates of PMH total and domain-specific scores within the patient population

After including the socio-demographic variables and diagnostic groups as predictors in the linear regression analyses, age correlated with total PMH and all other domains except for the interpersonal skills and emotional support domains. Ethnicity remained a significant predictor for the Spirituality domain and correlated with total PMH and general coping. In addition, gender was associated with emotional support and marital status with emotional support and interpersonal skills. Education status was significantly associated with total PMH and interpersonal skills while employment status correlated with total PMH, general coping, interpersonal skills and personal growth and autonomy. Significant associations between diagnostic groups and total PMH, general coping, emotional support, personal growth and autonomy and global affect remained, and in addition Spirituality also correlated with the diagnostic groups (Table 3).

**Table 3:** Significant Socio-demographic and clinical correlates of PMH total and domain scores

	Total PMH Score		General coping		Emotional support		Spirituality		Interpersonal skills		Personal growth and autonomy		Global affect	
	β	95 % CI	β	95 % CI	β	95 % CI	β	95 % CI	β	95 % CI	β	95 % CI	β	95 % CI
Age 21 to 39 years vs Age 40 to 65 years	−0.35**	(−0.56, −0.14)	−0.46**	(−0.71, −0.21)	−0.07	(−0.37, 0.23)	−0.58**	(−0.90, −0.26)	−0.19	(−0.42, 0.05)	−0.53**	(−0.78, −0.28)	−0.30*	(−0.56, −0.03)
Male vs Female	−0.15	(−0.35, 0.04)	0.02	(−0.22, 0.25)	−0.36*	(−0.64, −0.08)	−0.24	(−0.54, 0.05)	−0.07	(−0.28, 0.15)	−0.08	(−0.32, 0.15)	−0.18	(−0.42, 0.07)
Malay vs Chinese	0.34**	(0.10, 0.58)	−0.34*	(0.05, 0.62)	0.02	(−0.32, 0.36)	1.05**	(0.68, 1.42)	0.17	(−0.10, 0.43)	0.33*	(0.04, 0.62)	0.15	(−0.15, 0.46)
Indian vs Chinese	0.20	(−0.04, 0.43)	0.30*	(0.01, 0.58)	−0.25	(−0.59, 0.09)	0.59**	(0.22, 0.95)	0.09	(−0.18, 0.35)	0.33*	(0.04, 0.62)	0.13	(−0.18, 0.43)
Married vs Single	0.12	(−0.12, 0.35)	0.02	(−0.26, 0.29)	0.35*	(0.02, 0.68)	−0.04	(−0.40, 0.31)	0.29*	(0.03, 0.54)	0.14	(−0.14, 0.42)	−0.05	(−0.35, 0.25)
Some formal/primary education vs Post grad	−0.72*	(−1.44, −0.01)	−0.81	(−1.66, 0.05)	−0.67	(−1.70, 0.35)	−0.85	(−1.95, 0.24)	−0.94*	(−1.73, −0.15)	−0.80	(−1.66, 0.07)	−0.27	(−1.19, 0.64)
Unemployed vs Employed	−0.22*	(−0.42, −0.02)	−0.37**	(−0.61, −0.13)	−0.11	(−0.40, 0.18)	−0.04	(−0.35, 0.27)	−0.36**	(−0.58, −0.14)	−0.28*	(−0.52, −0.04)	−0.19	(−0.45, 0.06)
Depressive disorders vs Schizophrenia spectrum disorders	−0.54**	(−0.75, −0.32)	−0.45**	(−0.72, −0.19)	0.58**	(−0.89, −0.27)	−0.66**	(−0.10, −0.33)	−0.14	(−0.38, 0.11)	−0.42**	(−0.69, −0.15)	−0.98**	(−1.23, −0.73)
Anxiety disorders vs Schizophrenia spectrum disorders	−0.44**	(−0.69, −0.19)	−0.47**	(−0.78, −0.17)	−0.09	(−0.45, 0.27)	−0.94**	(−1.34, −0.55)	−0.02	(−0.31, 0.27)	−0.30	(−0.61, 0.02)	−0.82**	(−1.12, −0.52)

Relationship determined using General Linear Model

*P < =0.05, **P < =0.01

### Comparison of PMH and its domain mean scores between patient and general populations

The PMH instrument was administered on a multi-ethnic Asian population comprising of residents aged 21 to 65 years residing in households across Singapore in a study by Vaingankar et al. [12]. The PMH total mean score reported by the general population was 4.53, significantly higher than the overall patient population which reported a total mean PMH score of 3.94 (*p <* 0.001). One-sample t-tests showed significantly higher domain mean scores in the general population when compared to the diagnostic groups (Table 4). A further look into the different diagnostic groups showed significant differences across the populations. Except for general coping, spirituality and global affect, which did not show significant differences, PMH total scores and other domain scores were significantly lower in the schizophrenia spectrum patient population as compared to the general population. Significantly lower scores were observed for total PMH and the 6 domains for those with anxiety and depressive disorders in comparison with the general population. The overall patient population showed lower significant mean scores for across PMH and its domains.

**Table 4:** Comparison of total PMH and domain scores between general population and psychiatric populations

	df	Total PMH Score	*t*	General Coping	*t*	Emotional support	*t*	Spirituality	*t*	Interpersonal skills	*t*	Personal growth and autonomy	*t*	Global affect	*t*
General population		4.53		4.34		4.80		4.29		4.69		4.64		4.37	
Overall patient population	359	3.94	−11.90*	3.84	−8.44*	3.95	−12.07*	3.98	−4.06*	4.25	−8.02*	3.94	−11.62*	3.67	−11.32*
Schizophrenia Spectrum Disorders	141	4.24	−3.86*	4.11	−2.50^a^	4.20	−5.77*	4.44	1.42^b^	4.31	−4.30*	4.16	−5.18*	4.23	−1.62^c^
Depressive Disorders	138	3.70	−10.62*	3.66	−7.29*	3.62	−10.23*	3.78	−3.97*	4.17	−5.76*	3.75	−9.29*	3.25	−12.35*
Anxiety Disorders	78	3.80	−6.81*	3.64	−5.13*	4.11	−4.69*	3.49	−4.52*	4.29	−3.70*	3.87	−5.70*	3.41	−7.15*

*P < 0.001

^a^ *p* = 0.140 ^b^ *p* = 0.157 ^c^ *p* = 0.107

## Discussion

In this study we 1) explored the PMH levels in a multi-ethnic outpatient population, 2) examined the socio-demographic correlates of PMH across the various diagnostic groups as well as 3) observing the differences in the levels of PMH between patients with mental illnesses and the general population.

### Findings of the socio-demographic correlates of PMH among the diagnostic groups

(i) *Effect of age:* The older patients (40–65 years) reported higher levels of total PMH, general coping, spirituality and personal growth and autonomy. This has been similarly observed in other studies [13, 14] where it has been reported that older individuals are more psychologically mature than their younger counterparts, thus leading to increased personal growth. An increase in spiritual involvement in older age has been predicted by religious participation and their personality characteristics in early adulthood and following experiences of negative life events [15] which may explain why older patients scored higher in the spirituality domain. The effect of age remained significant for the aforementioned domains after controlling for other socio-demographic factors. In addition, global affect was found to be significantly correlated with age. This age effect in patients is consistent with the finding in the general population in Singapore where the older individuals had higher spirituality. However, this effect in the general population did not remain significant after controlling for other socio-demographic factors [12].
(ii) *Effect of gender:* Gender remained a significant predictor for emotional support, with females having a higher emotional support as similarly observed in the general population. In the general population however, personal growth and autonomy emerged as a significant domain as well [12]. Studies have shown that females report higher average levels of well-being than men [16, 17]. This difference between the genders can be attributed to their social roles which differ in terms of emotional experiences. Females tend to take on more of a ‘caretaker role’, hence are more likely to be sensitive to others’ needs and feelings. Males on the other hand are less likely to have emphasis on emotional experience. Typically females are known to be more emotionally expressive and sensitive [17].
(iii) *Effect of ethnicity:* There were significant ethnic differences with Malays and Indians having higher scores for the domains general coping, personal growth and autonomy and spirituality. Malays had a significant higher score in total PMH when compared to the Chinese. Studies have shown that culture and religion play a part in mental health [18, 19]. The differences within the ethnic groups in this study can be associated with religious identity which is the strongest in the Malay community followed by the Indians [20]. Intrinsic religiousness is known to have a positive effect on mental health, significantly predicting life satisfaction [21].
(iv) *Effect of marital status:* Married respondents reported higher levels of emotional support and interpersonal skills as compared to those who were single. Wood, Rhodes & Whelan [17] discuss that married individuals tend to report lower rates of psychological symptoms and seek psychological services less frequently when compared to the unmarried. They further suggest that marriage represents an increase in roles for men and women and this ‘multiple-role occupancy’ gives positive consequences on well-being and by extension, a married individuals’ PMH.
(v) *Effect of education:* Education level was significantly associated with total PMH and interpersonal skills. Keyes et al. [4] identified that education contributes to an individual’s psychological and subjective well-being. Michalos [22] highlighted that education has an impact on the eudaimonic aspect of well-being and our results support these previous findings.
(vi) *Effect of employment:* Employed respondents had higher total PMH, general coping and interpersonal skills domain scores. Unemployment can impair an individual’s psychological well-being and the impact of unemployment on mental health can manifest in multiple domains [23]. Employment can provide wealth and absence of wealth seems to create unhappiness [24]. It is important for an individual to be satisfied with his or her socio-economic status to have an overall satisfaction with life. Further research is needed to study the cause and effect relationship between the domains and employment status.

### Differences in PMH and its domain mean scores between the patient and general populations

The study has shown that patients belonging to the different diagnostic groups (schizophrenia spectrum disorders, depressive and anxiety disorders) in general have lower PMH and domain mean scores when compared to the general population. This suggests that the patient population has a lack of protective factors and mental health professionals can work on finding out the domains that are impaired for the individual patients. Existing literature reports that stigma related to mental illness harms the self-esteem of people with mental illnesses [25] and poor self-esteem is associated with a broad range of mental disorders and social problems [26]. The presence of a mental illness could predispose patients to have lower levels of PMH due to poor self-esteem and the impairment of the various domains.

i. *Spirituality:* Koenig [27] suggested that spirituality and mental health have a robust relationship as spirituality encompasses psychological, social, and behavioral aspects that are related to mental health. It is expected that spirituality boosts positive emotions and aids in neutralizing negative emotions, and therefore “serves as both a life-enhancing factor and as a coping resource”.
ii. *General coping:* A study by Aldwin and Revenson [28] found that those who had poorer mental health and more stress used less adaptive coping strategies which is in line with our findings in the domain of general coping.
iii. *Interpersonal skills:* Research indicates that the impairments in social functioning are strongly related to deficits in interpersonal skills, particularly in individuals with schizophrenia [29]. A study by Bowie et al. [30] showed that negative symptoms interfered with interpersonal relationships and depression imposed a limit on interpersonal functioning regardless of other competence domains. This explains the lower scores on the domain of interpersonal skills among the diagnostic groups. On the other hand, studies have shown that individuals with schizophrenia exposed to social skills training showed significantly greater acquisition and durability of social skills [31]. These findings of the various domains mentioned thus far highlight the importance of focusing on enhancing a particular domain; as by doing so it improves the overall PMH of the individual.
iv. *Emotional support:* It has been widely discussed that emotional support plays a pivotal role in one’s psychological well-being. Barnett and Gotlib [32] discuss that interpersonal dependency is related to mental health as studies have shown that “formerly depressed people report higher-than-normal levels of interpersonal dependency”, suggesting that remitted patients tend to be dependent on positive emotional support to maintain their self-esteem, thus it is critical to assess this domain more in depth for patients with depressive disorders.
v. *Personal growth and autonomy:* A eudaimonic concept of well-being; it displays an individual’s level of confidence and the ability to make decisions. One with a strong sense of purpose in life and having the ability to self-evaluate is expected to indicate a high level of PMH [12]. Again it becomes essential to take a domain-specific approach to address the overall positive mental health of an individual. With the presence of a mental illness, individuals could lose the sense of personal responsibility.
vi. *Global affect:* Diener et al. [33] discuss the ‘positive mood offset phenomenon’ which is when people experience positive moods when significant negative stimuli are absent. This mild to moderate positive mood is described as the automatic baseline in humans. On the other hand, the absence of positive moods results in mental health issues. The absence of positive mood offset seen in mood-related disorders can create problems in virtually all areas of the afflicted person’s life and be more debilitating a physical illness.

### Clinical implication

Our study findings emphasize the multi-dimensionality of mental health and explain the importance of assessing the domains of PMH in patients as that will aid in developing effective interventions for the patients. By assessing the PMH domains in patients, a mental health professional will be able to better comprehend the state that an individual might be undergoing- thriving/flourishing or surviving/languishing. It will also be discernible as to which precise domains the patients need strengthening in and that will allow for the symptoms to be properly addressed. This can lead to the patients to thrive by reducing the symptoms of the illness if not reducing the severity of the mental illness.

### Limitations

There are several limitations to this study. Firstly, as this is a cross-sectional study we are unable to establish any causal relationships. Secondly, only English speaking adults were included in the survey which may bias the sample. Lastly, we could not collect information on non-response rates due to the convenience sampling strategy and recruitment at various sites.

## Conclusion

It is essential for mental health professionals to examine the domains of every patient’s positive mental health. By focusing on the domains where the patient might have a deficit, they will be able to implement appropriate interventions and treatment strategies to promote the patients’ positive mental health. Promoting positive mental health will complement the approach of treatment and risk reduction for improving national mental health.

## Abbreviations

ES: Emotional support
GA: Global Affect
GC: General coping
IS: Interpersonal skills
PGA: Personal growth and Autonomy
PMH: Positive mental health
